# Retraction: Regulation of gastric smooth muscle contraction via Ca^2+^-dependent and Ca^2+^-independent actin polymerization

**DOI:** 10.1371/journal.pone.0225159

**Published:** 2019-11-07

**Authors:** The PLOS ONE Editors

After publication of this article [1], the corresponding author requested its retraction. They noted concerns about Figs 2–5, claimed that mistakes had been made in preparing figures such that data were errantly reused, and raised that some of the underlying data for the reported results are no longer available. The authors indicated that they are confident in the results and conclusions reported in the article.

In reviewing the results, we noted the following concerns:

- Lanes 2–4 of the RhoA panel in Fig 2A appear similar to lanes 1–3 of the FAK panel in Fig 2B.
- In Fig 3B, the Basal and ACh +PF573228 panels appear similar.
- The IP: Paxillin panels in Fig 4D, 4E and 4F appear similar, when horizontally flipped and/or adjusted for aspect ratio.
- The RhoA [T19N] lanes of the IP: Paxillin panel in Fig 4E appear similar to lanes 2, 3 in the RhoA panel of Fig 2A.
- Lanes 1, 2, 5 in the IP: N-WASp panel in Fig 5B are similar to lanes 5, 4, 1, respectively, in the lower blot of Fig 4F, when lanes 1, 2 in the Fig 5B panel are flipped horizontally.
- There is an abrupt horizontal discontinuity at the bottom of the band in lane 2 of the p-Paxillin blot in Fig 15.
- The quality of blot images in multiple figures is not sufficient to verify the integrity of the data.

The authors confirmed that the Fig 3B panels were duplicated in error but did not comment on the other concerns or clarify which data are no longer available. As such, we cannot clarify the issues or confirm the reliability of the results.

In light of these issues, the *PLOS ONE* Editors retract the article.

All authors agree with the retraction.
